# Influence of Salting and Ripening Conditions on the Characteristics of a Reduced-Fat, Semi-Hard, Sheep Milk Cheese

**DOI:** 10.3390/foods12244501

**Published:** 2023-12-16

**Authors:** Lambros Sakkas, Ekaterini Moschopoulou, Golfo Moatsou

**Affiliations:** Laboratory of Dairy Research, Department of Food Science and Human Nutrition, Agricultural University of Athens, Iera Odos 75, 118 55 Athens, Greece; lasakkas@hotmail.com (L.S.); catmos@aua.gr (E.M.)

**Keywords:** cheese ripening, reduced-fat sheep milk cheese, reduced-salt cheese, salting method, meltability, proteolysis, organic acids, texture profile analysis, bacterial groups

## Abstract

This study aimed to assess the effect of salting and ripening conditions on the features of sheep milk, reduced-fat, semi-hard cheese. Eight groups of cheese, with an average fat content of ≅10.5%, moisture on non-fat substances (MNFS) ≅ 56%, a protein-to-fat ratio of 2.9 and pH 5.1, were manufactured and analyzed throughout ripening. The experimental factors were the salting method (brine- or dry-salting), the salt content (control- and reduced-salt) and the ripening temperature sequence (11 or 18 °C at the 3rd and 4th week). Brine-salted cheese exhibited significantly more adequate (*p* < 0.05) textural and organoleptic characteristics compared to its dry-salted counterpart, i.e., lower hardness, gumminess and adhesiveness, with higher lightness and flavor scores. The mean salt reduction from 2.1 to 1.6% exhibited significant effects (*p* < 0.05), i.e., increased moisture and MNFS, decreased hardness, gumminess, chewiness and adhesiveness, and increased lightness and meltability of cheese without affecting the microbiological stability or impairing the organoleptic parameters. Ripening at 18 °C at weeks 3–4 significantly increased (*p* < 0.05) proteolysis and concentrations of lactic and citric acid without affecting meltability, textural or organoleptic features. In conclusion, brine-salting, salt reduction by 20% and the elevation of temperature at a particular ripening period improved the characteristics of this type of reduced-fat sheep milk cheese.

## 1. Introduction

Following the suggestions of the World Health Organization (WHO) for the reduction of fat and sodium intake from food [1,2], there are constant research efforts for the development of reduced- or low-fat and -sodium cheese and the improvement of their quality. Interventions in regard to both these cheese substances are a great challenge for the complex cheese manufacturing steps and for the properties and shelf-life of the final products.

The reduction of fat content of cheese usually coincides with reduced organoleptic scores and changes in the sensory and functional attributes. There is an array of phenomena that are affected by the fat reduction. In brief, substrates for lipolysis and the subsequent formation of flavor and volatile compounds are reduced. Fat is a very crucial element of the cheese microstructure and mechanical/rheological properties because its dispersion within the paracasein network prevents the local aggregation of caseins. The ratio of protein to fat affects the degree of primary (breakdown of caseins into large and medium-sized peptides) and secondary (further breakdown of peptides into smaller peptides and amino acids) proteolysis during cheese ripening. The action of fat as a “filler” in the paracasein network results in “lubrication” at the microstructural level and contributes to the development of a smooth and creamy texture that can melt and spread upon heating. In addition, fat contributes indirectly to the release rate of flavor/aromatic substances during chewing, due to its effect on the structural and rheological characteristics of cheeses [3,4,5,6]. The increase in moisture in such a way that moisture on non-fat substances (MNFS) is similar to that of the full-fat counterpart and an increase in the proteolysis level can counteract the loss of yield, the increase in firmness and the decrease in organoleptic scores in reduced/low-fat cheese. A consequence of the increase in moisture and thus in the residual lactose is the increase in acidity in this type of cheese [4,6,7]. To address these problems, a variety of modifications have been suggested for the treatment of cheese milk and for the cheesemaking conditions. The application of curd washing to limit the development of acidity and the increase in ripening temperature to enhance proteolysis have been utilized as experimental factors in the present study. The increase in ripening temperature can increase the level of secondary proteolysis; however, the high moisture content may cause excessive growth of non-starter lactic acid bacteria (NSLAB), and the development of off-flavors may be observed [4,6].

The effect of salt reduction on the characteristics of reduced-fat cheese was also studied in the present study. Salt is an indispensable ingredient of cheese and a decisive factor for the quality of all cheese types, since it affects their organoleptic, structural and physical properties, as well as their stability. Its effect depends on its concentration in the liquid phase of the cheese, expressed as salt in moisture (S/M) that fluctuates within an optimal range for each cheese variety. S/M is a major determinant of the microbial growth and enzymatic activities occurring during ripening by reducing and controlling water activity (a_w_). Moreover, salt enhances water binding by caseins, thus affecting the solubility and interactions of the protein molecules and the rheological, structural and functional properties of cheeses during heating. Finally, in the minds of consumers, salt is traditionally linked to the organoleptic character of cheese [4,5,6,7,8,9,10,11]. Due to the above, there are several difficulties in the successful production of reduced/low-salt cheese. These cheese types typically exhibit higher moisture content, higher a_w_, and higher growth of desirable and undesirable bacteria. Intense bacterial growth, combined with uncontrolled enzyme activity, increases proteolysis, especially the quantity of small—often bitter—peptides, and increases lactose fermentation, which in turn reduces pH. Although there may be a higher concentration of free amino acids and volatile aromatic compounds, the characteristic flavor of a particular reduced-salt cheese variety is less intense or even absent, the texture is softer and weaker and the melting capacity and fluidity are higher compared to its normal-salt counterpart [4,8,9].

The aim of the present study was to assess the effect of salting and ripening conditions on the features of sheep milk, reduced-fat and reduced-salt semi-hard cheese. The experimental interventions were: (a) the salt content of the cheeses, (b) the salting method and (c) the ripening temperature sequence. Eight types of reduced-fat semi-hard sheep experimental cheeses with an average protein/fat ratio of ≈2.9 were manufactured. The presentation, discussion and assessment of the findings was based on the following: i. various types of analyses carried out throughout a 16-week ripening and storage period; ii. the results of statistical analysis.

## 2. Materials and Methods

### 2.1. Cheesemakings

Cheese milk was standardized by mixing whole raw and skimmed sheep milk at a ratio of 0.47 ± 0.05. Skimmed milk was prepared from raw sheep milk preheated at 50 °C using a cream separator (Janschitz GMBH, Althofen, Austria). The pH of raw and skimmed milk was 6.56 ± 0.08 and 6.54 ± 0.04, respectively. The cheese milk mixture contained 5.20 ± 0.01% protein and 1.66 ± 0.01% fat, which corresponded to a protein-to-fat ratio (P/F) of 3.14 ± 0.03.

**Figure 1:**
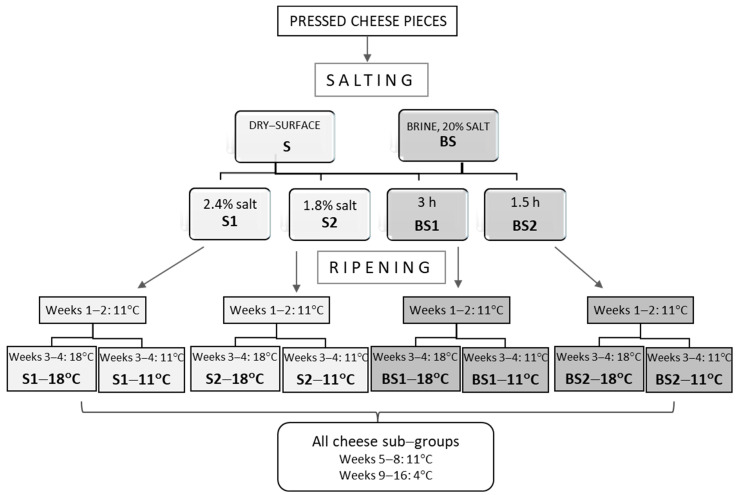
Salting, ripening and coding of experimental cheese groups.

The standardized cheese milk was pasteurized at 68 °C for 10 min, immediately cooled down at 32 °C and inoculated with the commercial starter CHOOSIT AlpD (Danisco, DuPont Nutrition & Biosciences, Copenhagen, Denmark). The starter was a mixture of *Lactococcus lactis* subsp. *lactis*, *Lactococcus lactis* subsp. *cremoris*, *Lactococcus lactis* subsp. *lactis* biovar. *diacetylactis*, *Streptococcus thermophilus*, *Lactobacillus helveticus* and *Lactobacillus lactis*. After five minutes, the commercial rennet powder Naturen Extra 1125 NB (Chr. Hansen, Hørsholm, Denmark) was added at a ratio of 4 g per 100 kg milk. Cheese curdling was performed at 32 °C within 36.2 ± 2.1 min after the addition of rennet and then the cheese curd was cut into cubes with an edge of 1–1.2 cm. After stirring the cheese curd pieces for 15 min at 35 °C, a part of whey was removed and replaced by water heated at 50 °C. Then, scalding was carried out up to 38 °C for 30 min. Curd was collected into rectangular molds with the aid of cheesecloths and was pressed for 40 min, applying a weight equal to curd weight. The pressed curd was cut into cheese pieces of approximately 12 × 11 × 5 cm that were held overnight at 11 °C. Thereafter, different salting and ripening conditions were applied as summarized in Figure 1.

Cheese pieces were divided into two groups. The first group was dry-surface salted cheeses coded as “S”. They were salted at 11 °C by depositing coarse salt on the upper and lower surfaces of the cheese at a total ratio of 2.4% or 1.8% (*w*/*w*), thus resulting in two subgroups coded as “S1” (control-salt dry-salted cheese) and “S2” (reduced-salt dry-salted cheese). The second group, which was coded as «ΒS», of cheese was salted in a brine containing 20% NaCl and 0.3% CaCl_2_ (*w*/*w*), which was coded as «ΒS». The duration of salting was 3 ± 0.07 or 1.50 ± 0.02 h, resulting in two subgroups coded as “BS1” (control-salt brine-salted cheese) and “BS2” (reduced-salt brine-salted cheese). Subsequently, all cheese sub-groups remained at 11 °C, regularly overturned.

On the 8th day, after the assessment of pH and of gross composition, they were vacuum packed and remained for another 5 days at 11 °C. Then, half of the cheeses of each sub-group were transferred to a different ripening room at 18 °C, where they remained until the end of 4th week. Thus, 8 sub-groups of experimental cheeses were obtained based on the experimental factors, i.e., salting method × salt level × ripening scheme. Three repetitions were carried out for each sub-group. Cheese codes are depicted in Figure 1. From the 5th to the end of 8th week, all sub-groups remained at 11 °C and then they were transferred to storage room at 4 °C.

### 2.2. Analyses

Fat contents of milk and whey were determined according to the Gerber method [12]. Fat content of cream was determined in a mixture of cream and distilled water at a 1:1 ratio by means of the specially designed “Koehler” butyrometer. Gross composition of milk, mixtures and whey was determined by Milkoscan-FT120 (Foss, Hillerød, Denmark) and pH by means of a pH meter.

Cheese pH was evaluated in a dispersion of cheese in distilled water at a 1:1 ratio. The gross composition of cheese was determined by FoodScan-Dairy Near Infrared (NIR) analyzer (Foss, Hillerød, Denmark). In addition, cheese moisture was determined in triplicate according to the oven method [13]. The salt content of cheese was determined in duplicate by potentiometric titration [14].

The evaluation of proteolysis was based on the assessment of free amino-groups by the trinitrobenzensulfonic acid (TNBS) method described by Moschopoulou et al. [15] with two modifications, i.e., the aqueous dilution of the supernatant was 6% (*v*/*v*) and the stopping solution was 0.1 N HCl. The results were calculated by means of a standard curve and were expressed as mM glycine (mM gly).

The analysis of the parameters of texture profile was based on the first and second bite force distance curves [16] and was carried out according to Moatsou et al. [17]. Cheese color (L*, a*, b* values) was measured by the LC Spectrocolometer (Lovibond, Dortmund, Germany). The Schreiber test described by Park et al. [18] was used for the assessment of meltablity with the modifications reported by Sakkas et al. [19]; one cm of mean cheese expansion corresponded to 2 meltability units.

Bacterial counts were estimated at 4 and 16 weeks of ripening by means of the colony count technique [20]. The growth of mesophilic cocci was performed in M17 agar medium, pH 6.8, incubated at 30 °C for 48 h. For the non-starter lactic acid bacteria, a double-layer of Rogosa agar pH 5.4 was used and incubation was carried out at 30 °C for 5 days.

Residual sugars and organic acids of cheese were determined by the HPLC method described by Lepesioti et al. [21] using an Aminex HPX-87H column (Biorad Inc., Hercules, CA, USA). Solvent was 5 mM H_2_SO_4_ and flow rate was 0.5 mL/mim, at 35 °C. Detection was carried out by means of refractive index (RI Detector FXRIDet-3 1:1, Flexar, PerkinElmer Inc., Shelton, WA, USA) and UV absorbance (PDA Detector 210:10:400:10:1, Flexar, PerkinElmer Inc., Shelton, WA, USA). The sample preparation was modified as follows. A mixture consisted of 5 g cheese and 5 g ultrapure water were diluted with 20 mL of a sodium tungstate solution (0.7 g sodium tungstate dihydrate, 10 μL of an 88% solution of orthophosphoric acid and 7 mL of 1N sulfuric acid adjusted to 100 mL with ultrapure water) and 20 mL ultrapure water. After stirring and a 10 min resting period, the cheese solution was filtered using a fast-speed filter paper. One milliliter of the filtrate was thoroughly mixed with 0.1 mL of a 70% perchloric acid solution and remained at 4 °C overnight. Then, it was centrifuged at 13,000× *g* for 60 min at 4 °C and the supernatant was filtered with 0.22 μm syringe filter before HPLC analysis.

Organoleptic parameters of cheeses at weeks 8 and 16 were examined by a panel of six experienced laboratory staff members, who signed an Informed Consent document for the organoleptic assessment of cheese samples coded using three-digit codes, which were randomly presented to the panel for the evaluation of appearance, texture, and flavor using a scale from 0 to 10 points. The total organoleptic score—expressed as a percentage—was the sum of the appearance, texture, and flavor scores multiplied by 1, 4, and 5, respectively [19,21]. Moreover, the panel members noted the existence of specific textural and flavor features and defects.

### 2.3. Statistical Analysis

The effects of the stage of ripening, the means of salting, the salt content and the ripening conditions, and their interactions were assessed by multifactor ANOVA. The least significance (LSD) method (*p* < 0.05) was applied to test the significant differences. Analysis was performed by means of Statgraphics Centurion XVI (Manugistics, Inc., Rockville, MA 20852, USA). The results of the statistical analysis and in particular the statistically significant effects (*p* < 0.05) were taken into consideration for the configuration of the tables and the discussion of the findings.

## 3. Results

### 3.1. Physicochemical Composition

**Figure 2:**
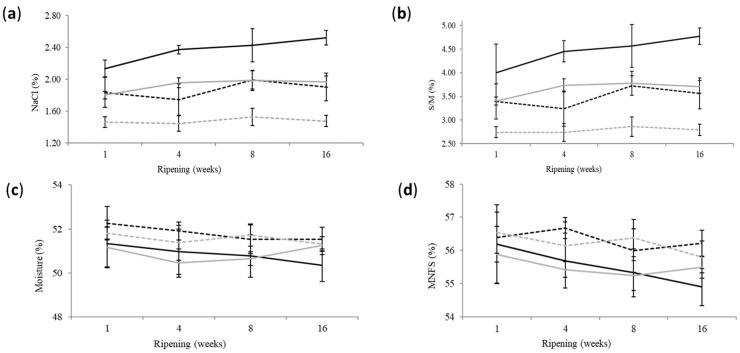
Evolution of salt content (**a**), salt-in-moisture symbolized as S/M (**b**), moisture (**c**) and moisture on non-fat substances symbolized as MNFS (**d**) during the ripening and storage of reduced-fat sheep milk cheese. Error bars: standard deviation. Black-solid line: dry-salted control-salt cheese (S1); Black-dashed line: dry-salted reduced-salt cheese (S2); Gray-solid line: brine-salted control-salt cheese (BS1); Gray-dashed line: brine-salted reduced-salt cheese (BS2).

The stage of ripening, the salting method and the salt quantity had a statistically significant effect (*p* < 0.05) on most of the compositional parameters, whereas the ripening temperature did not affect them (Table 1, Figure 2). Moisture on non-fat substances (MNFS) of all cheese groups was within the range from 54 to 69% reported by Codex Alimentarius [22] for semi-hard cheese. The same holds true for fat on dry matter (FDM), which was within the range from 10 to 25%, corresponding to partially skimmed cheese [22].

The mean salt content of S1 and S2 cheeses was 2.36 ± 0.224% and 1.87 ± 0.184%, respectively. The respective content of brine-salted cheese was 1.93 ± 0.108% and 1.48 ± 0.08%. The salt content of S2 and BS2 cheese was 21 and 23% lower than that of S1 and BS1 control-salt counterparts, respectively. The sodium content of S1 and S2 cheese was 0.93 and 0.74%, higher than the respective 0.76 and 0.58% of BS1 and BS2 cheese. Therefore, BS2 cheese was very advantageous in terms of the strong recommendation of the World Health Organization for a reduction of salt intake to <2 g/day sodium or 5 g/day salt [2].

The means of salting influences the rate of salt intake in the cheese mass. A retardation of salt intake is expected for dry-salted cheese. At first, a part of salt is diluted in the moisture of the cheese surface and, as it diffuses in the interior of cheese, it causes whey expulsion. In turn, the expelled whey dilutes more salt, thus creating a saturated brine that may induce intense moisture loss from the cheese rind, which retards further salt intake [11]. However, the high ratio of surface to volume of the cheese pieces of the present study favored the dry salt intake.

The salting method (S or BS) significantly affected (*p* < 0.05) the cheese pH, but the salt quantity did not. BS cheese had lower pH and higher acidity compared to the S counterparts, which can be attributed to lower salt and S/M contents. As discussed previously, salt intake was faster in fresh dry-salted cheese, which in turn retarded the fermentation of residual lactose [8,9]. In conclusion, within the salt levels and conditions of the present study, the rate of the salt intake and not the quantity controlled the acidification of the cheese mass.

The means of salting (S or BS) did not statistically significantly affect the moisture and MNFS contents of cheese; the opposite was true for the salt quantity. Moisture and MNFS contents of reduced-fat S2 and BS2 cheese were significantly higher than those of control-salt counterparts, resulting in the lower protein content of the former. Despite the significantly lower protein content (*p* < 0.05), reduced-salt cheese had significantly (*p* < 0.05) higher PDM content compared to control-salt counterparts. Fat and FDM contents were significantly (*p* < 0.05) affected by the means of salting, being higher in BS cheese, but the salt quantity did not have a significant effect on their values. However, P/F ratio was not significantly (*p* > 0.05) affected by the salt content and the means of salting (Table 1).

The gross composition of cheese was affected significantly by the ripening time but not by the ripening temperature scheme. The ripening time decreased moisture, MNFS, fat, FDM, protein and PDM contents and increased salt and S/M contents and P/F ratio. Most of the significant differences (*p* < 0.05) were observed in the interval between week 1 and week 4, whereas no significant differences were found thereafter up to week 16. The evolution of salt and S/M contents (Table 1, Figure 2) followed the evolution of moisture. A significant increase was (*p* < 0.05) observed at week 8, without any significant changes thereafter. Interestingly, moisture change was very limited, i.e., 0.5%, due to the fact that cheese ripened under packaging. The retention of moisture is a major aspect of the manufacture of reduced-fat cheese [3,4,5,6,7].

In contrast to the stability of cheese pH during ripening, the titratable acidity increased significantly (*p* < 0.05). The pH expresses the change of H^+^, while titratable acidity depends on the buffering capacity in the range from the actual cheese pH to pH 8.3, at which the color change of the titration index occurs [23]. The buffering capacity of cheese is configured by the products of bacterial metabolism and their changes occurring during ripening. In brief, the cheese components that contribute largely to the cheese buffering capacity are caseins, and their hydrolysis products and the concentration and distribution of minerals and organic acids such as lactic, citric, propionic, acetic and butyric acids [24]. The non-significant effect of ripening on cheese pH indicates that the increase in organic acids (presented in Section 3.6) was counteracted by the increase in buffering capacity. The high protein content of the reduced-fat cheese of the present study contributes to the increase in buffering capacity. Moreover, increase in the buffering capacity of Cheddar and Emmental cheese in the range of pH 5–6 during ripening has been reported due to the solubilization of colloidal calcium phosphate evolving in parallel to proteolysis [24,25,26].

### 3.2. Proteolysis

**Figure 3:**
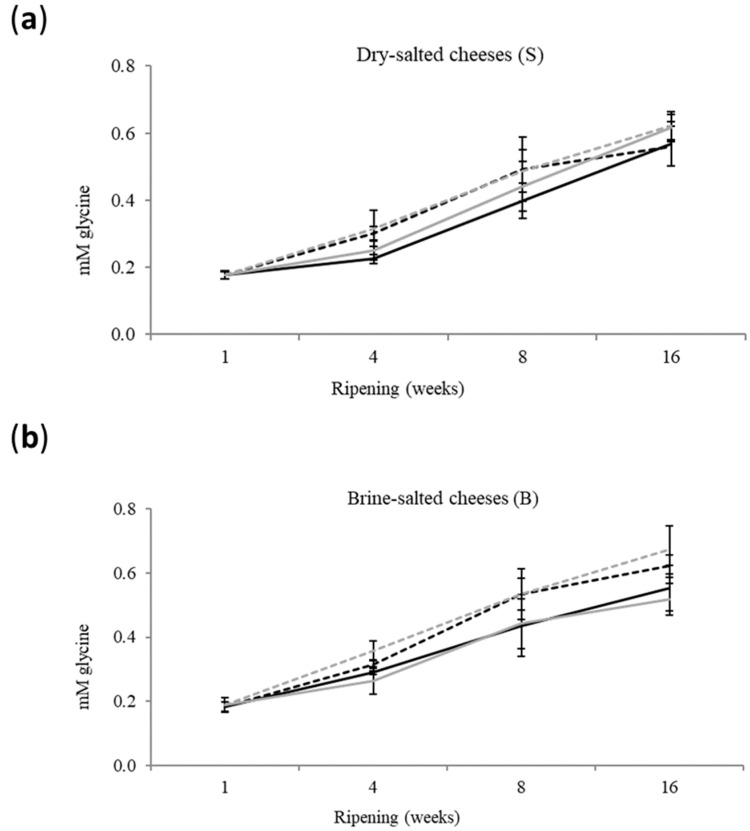
Evolution of proteolysis expressed as free amino groups (mM glycine) of dry-salted (**a**) and brine-salted (**b**) during the ripening and storage of reduced-fat sheep milk cheese. Error bars: standard deviation. Black-solid line: control-salt cheese ripened at 11 °C at the 3rd and 4th week; Black-dashed line: control-salt cheese ripened at 18 °C at the 3rd and 4th week; Gray-solid line: reduced-salt cheese ripened at 11 °C at the 3rd and 4th week; Gray-dashed line: reduced-salt cheese ripened at 18 °C at the 3rd and 4th week.

Τhe level of proteolysis of cheese (Figure 3) was significantly affected (*p* < 0.05) only by the stage and the conditions of ripening. No significant interactions of these factors with the salting parameters were observed. Indicatively, the average proteolysis level of 11 and 18 °C cheese was 0.358 ± 0.07 and 0.409 ± 0.007 mM gly, respectively. Apparently, the increase in temperature at the 4th week favored the enzymatic and bacterial activity [27].

In general, the increase in salt content does not favor the evolution of proteolysis by affecting the a_w_, MNFS, enzyme and microbial activity and water binding by caseins. However, these effects are limited, as the cheese pH decreases from pH 6.6 to 5.4 [9,11]. Therefore, the pH of the cheese of the present study—which was <pH 5.4—is expected to be related with the non-significant effect of the salt content on the proteolysis level.

### 3.3. Texture Profile Analysis

The stage of ripening, the salting method (S or BS) and the salt content significantly affected (*p* < 0.05) the hardness, cohesiveness, adhesiveness and gumminess. The ripening temperature sequence did not significantly affect (*p* > 0.05) the texture profile parameters. Elasticity and chewiness were affected (*p* < 0.05) solely by the stage of ripening.

**Figure 4:**
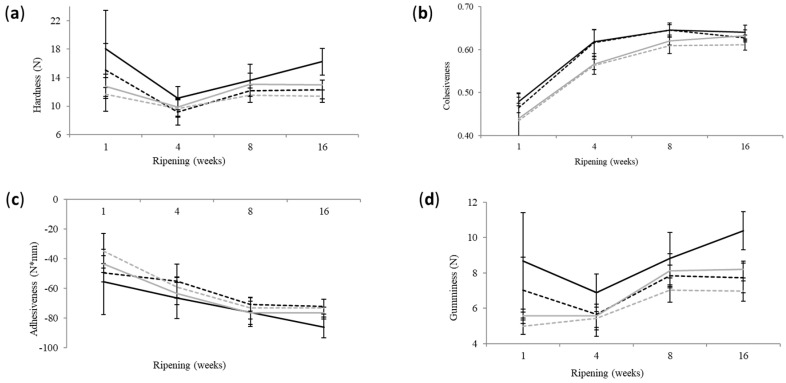
Evolution of textural parameters during the ripening and storage of reduced-fat sheep milk cheese. (**a**) Hardness, (**b**) cohesiveness, (**c**) adhesiveness, and (**d**) gumminess. Error bars: standard deviation. Black-solid line: dry-salted control-salt cheese (S1); Black-dashed line: dry-salted reduced-salt cheese (S2); Gray-solid line: brine-salted control-salt cheese (BS1); Gray-dashed line: brine-salted reduced-salt cheese (BS2).

In regard to the stage of ripening, the most intense changes were observed between the 1st and the 4th week. No significant changes occurred after week 8. Interestingly, a considerable increase in hardness was observed after the 4th week, although the moisture did not change (Figure 4). A similar behavior of Cheddar cheese has been attributed to the increase in proteolysis that results in free amino- and carboxyl-groups that in turn bind water [16,28].

BS cheese exhibited significantly lower (*p* < 0.05) hardness, cohesiveness and gumminess than the S counterpart. Moreover, the absolute value of adhesiveness was significantly (*p* < 0.05) lower in BS cheese, indicating that brine-salted cheese was less adhesive compared to dry-salted cheese. Most variables showed significant (*p* < 0.05) correlations with most composition parameters and proteolysis. According to those findings, cheese textural characteristics were improved by salting in brine.

The same holds true for reduced-salt compared to control-salt cheese. Hardness, gumminess, chewiness and adhesiveness were significantly (*p* < 0.05) lower in reduced-salt cheese. The reduction of salt influences texture parameters because it is related to cheese a_w_, pH, proteolysis and gross composition of cheese and the water binding by casein. The increased moisture of reduced-fat cheese results in a reduction of the density of the paracasein matrix. Salt increases the binding of water by the paracasein due to the substitution of colloidal calcium with sodium ions that bind water. This phenomenon is crucial for the cheese microstructure because it enhances the uniformity of the paracasein matrix. At lower salt levels, the structure of cheese softens and becomes weak, whereas hardness decreases [8,11,29,30,31]. All of the above are in accordance with the present findings. In conclusion, the reduction of the salt content and the salting in brine did not impair the parameters of cheese structure but decreased hardness, which is a desirable finding for reduced-fat cheese.

### 3.4. Meltability and Color

Cheese meltability (Table 2) was significantly affected (*p* < 0.05) by the stage of ripening and the salt content. A significant increase was observed in the interval from 8 to 16 weeks. The reduction of salt content increased meltability significantly (*p* < 0.05), whereas the method of salting and the ripening temperature sequence had no significant effect. In most of the relevant studies, the salt reduction coincides with an increase in meltability in accordance with the present findings. This effect has been assigned to higher a_w_, moisture and proteolysis levels of reduced-salt cheese compared to control-salt counterparts [8]. In the present study, the correlation of meltability with moisture and MNFS was significant (*p* < 0.05) but moderate, i.e., r = 0.408 and r = 0.446, respectively, but it was not significantly correlated with proteolysis. The increase in meltability in reduced-salt cheese has been assigned to the decrease in water binding in paracasein that, in turn, decreases its volume and increases the distance between its elements [8].

The L* parameter of color that expresses the lightness was significantly higher (*p* < 0.05) in reduced-salt compared to control-salt cheese (Table 2). The same was true for the BS compared to S cheese. The a* parameter was affected by the salting method, and it was higher—less green color—in brine-salted cheese. The b* parameter was affected only by the stage of ripening and increased—more yellow color—at week 16. The increase in lightness can be ascribed to more wide channels of free-whey within the paracasein matrix of the reduced-salt cheese due to the decrease in water binding by the paracasein. In this case, these channels act as light scattering regions [11]. The increased lightness observed in reduced-salt and brine-salted cheese is a desirable feature for reduced-fat cheese.

### 3.5. Bacterial Counts

**Figure 5:**
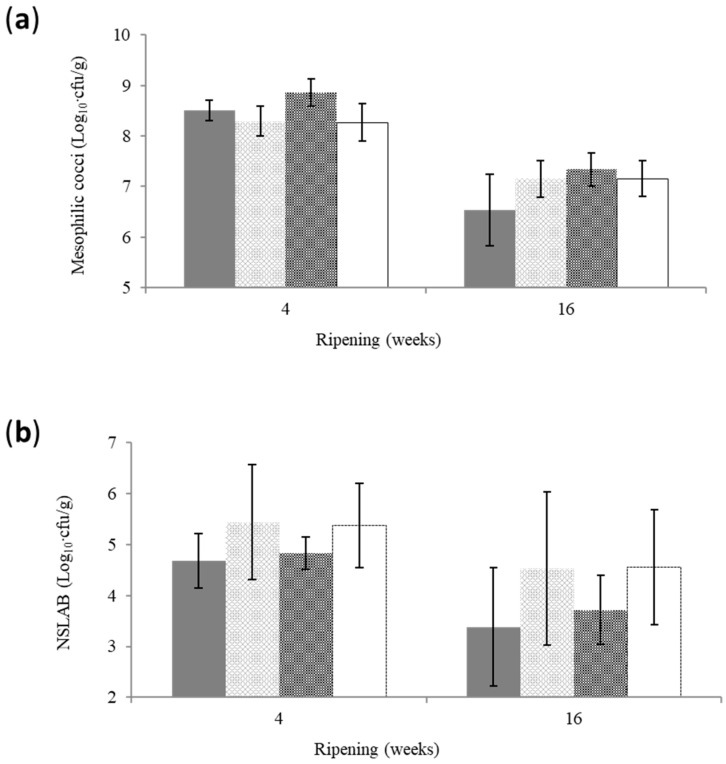
Evolution of counts of mesophilic cocci (**a**) and non-starter lactic acid bacteria (NSLAB) (**b**) during the ripening and storage of reduced-fat sheep milk cheese. Error bars: standard deviation. 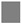 control-salt (S1, BS1)/11 °C at the 3rd and 4th week of ripening; 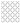 control salt (S1, BS1)/18 °C at the 3rd and 4th week of ripening; 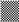 reduced salt (S2, BS2)/11 °C at the 3rd and 4th week of ripening; 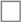 reduced salt (S2, BS2)/18 °C at the 3rd and 4th week of ripening.

The evolution of bacterial counts is depicted in Figure 5. The counts of mesophilic cocci were affected significantly (*p* < 0.05) by the stage of ripening and salt concentration. Despite the fact that the effect of ripening temperature sequence was not significant, the interactions of this factor with the stage of ripening and the salt content were significant. NSLAB counts were affected significantly (*p* < 0.05) by the stage of ripening and the ripening temperature sequence. The salting method did not affect these two bacterial groups. The mean mesophilic cocci count of reduced-salt cheese (S2, BS2) was significantly higher (*p* < 0.05) compared to the control salt counterpart (S1, BS1), i.e., 7.90 ± 0.771 vs. 7.62 ± 0.921 log_10_·cfu/g. Moreover, mesophilic cocci were significantly reduced from 8.48 ± 0.360 at week 4 to 7.05 ± 0.532 log_10_·cfu/g at week 16, on average.

Mesophilic cocci were included in the starter mixture (Section 2.1). This group grows and proliferates during the early stages of cheese ripening. Depending on cheese variety, their count decreases within the first weeks of ripening while the NSLAB counts increase substantially [10,32]. In general, salt impairs the growth of starter and NSLAB since it decreases a_w_ and increases the osmotic pressure of cheese moisture [9,11]. In particular, the growth of mesophilic cocci is favored when S/M content is within 2–3% and is obstructed thereafter [9]. The control-salt cheese of the present study contained on average 4.05 ± 0.534% S/M. The respective S/M content in the reduced-salt counterpart was 3.13 ± 0.441%, which coincided with higher mesophilic cocci counts. Similar findings have been reported for the semi-hard Samsoe cheese [31], whereas no significant effect of salt content has been reported for the semi-hard Roblochon [33].

The mean NSLAB counts were significantly reduced (*p* < 0.05) from week 4 (5.08 ± 0.790 log_10_·cfu/g) to week 16 (4.05 ± 1.195 log_10_·cfu/g). They were significantly higher in 18 °C compared to 11 °C cheese, i.e., 4.97 ± 1.177 vs. 4.15 ± 0.936 log_10_·cfu/g. The growth of NSLAB depends on their initial count, the strain, the rate of cooling of the cheese mass during pressing, the S/M and the ripening temperature [11]. The increase in the ripening temperature favors the growth of NSLAB [10,11], similar to the present findings.

The lack of significant effect of salt content on the NSLAB counts has also been previously reported [31], indicating that reduced-salt cheese was microbiologically stable. The reduction of salt of Cheddar cheese from 1.8 to 1.3% under similar moisture contents did not affect the starter and NSLAB counts and the acidification [34]. However, the opposite has been reported for the same cheese with similar salt contents [35].

### 3.6. Residual Sugars and Organic Acids

No residual lactose was found, indicating a rapid consumption by the starter bacteria. Mean values and statistical analysis results for galactose and various organic acids are shown in Table 3. The stage of ripening significantly affected (*p* < 0.05) all the variables. Salt content had a significant effect on galactose and lactic acid. The ripening temperature sequence significantly affected galactose, lactic acid and citric acid. Statistically significant interactions (*p* < 0.05) of the stage of ripening with other experimental factors in regard to the concentration of acetic acid were observed.

The organic acid content of cheese varies widely in the literature. Cheese of the present experiment contained 2300–2700 mg/100 g lactic acid. A lactic acid concentration of 1750–2750 mg/100g is reported for Gouda [36], 1720–1950 mg/100g for semi-hard Colby [37] and 1300–2400 mg/100g for mature Cheddar [38]. Citric acid content of the cheese of the present study was within 509–598 mg/100g. A citric acid content of 90–300 mg/100 g has been estimated in Gouda [36] and 15–550 mg/100g in Cheddar [39]. A low quantity of orotic acid has been found in Cheddar [39]. Formic acid concentrations of 10–110 and 53–220 mg/100 g have been reported for Gouda and Colby cheese, respectively [36,37]. A wide range of acetic acid content has been estimated during the ripening of different cheese varieties, such as, 70–220 mg/100 g for Gouda [36], 119–385 mg/100g for Colby [37] or 90–430 mg/100 g for Cheddar [38].

Despite the fast hydrolysis of lactose by the starters, galactose often is accumulated in considerable amounts in cheese since it is not metabolized by all starters. This holds true for *Streptococcus thermophilus* and *Lactobacillus lactis*, which were included in the starter mixture utilized in the present experiments. Galactose accumulated during the early phase of ripening may be catabolized later by the NSLAB [40,41]. Therefore, the significant reduction of galactose concentration during ripening (Table 3) is expected. Moreover, the decrease in salt content and the increase in ripening temperature (weeks 3 and 4) enhanced the catabolism of galactose.

The significantly higher (*p* < 0.05) concentration of lactic acid of 18 °C cheese compared to the 11 °C counterpart is in accordance to the significantly (*p* < 0.05) higher bacterial counts of the former. Nevertheless, this difference was not depicted in pH and titratable acidity due to the effect of buffering capacity on these analyses as previously discussed (Section 3.1). BS cheese contained significantly more lactic acid compared to the S group. Apparently, a slow diffusion of salt in the cheese mass increased the rate of the catabolism of lactose. As expected, ripening time and reduction of salt content significantly (*p* < 0.05) increased lactic acid concentration.

Citric acid levels remained steady up to 4 weeks of ripening, indicating that the bacteria of the starter mixture did not exhibit high citrate fermentation activity. A substantial metabolism of citrate by NSLAB could be expected. The NSLAB group usually includes *Lactobacillus* spp. strains with high citrate fermentation potential. However, in the present study, the increase in citric acid coincided with the increase in NSLAB count, i.e., at 18 °C or at week 8. A similar phenomenon observed during Cheddar or Gouda ripening has been connected to the metabolic activity of NSLAB or the increase in ripening temperature, respectively [36,39]. Increase in citrate has been also reported for Cheddar cheese with salt content from 0.8 to 1.3% [34]. After 4 weeks, citric acid concentration increased significantly (*p* < 0.05) and elevation of ripening temperature from 11 to 18 °C also significantly increased (*p* < 0.05) its levels.

The concentration of orotic and formic acids were steadily increased (*p* < 0.05) up to week 8. Relevant literature reports are inconsistent. Reduction of orotic acid and steady levels of formic acid during the ripening of Cheddar [39] and increase in formic acid during Gouda ripening [36] have been estimated. Salting method, salt content and ripening temperature did not significantly (*p* > 0.05) affect orotic and formic acid levels.

The concentration of acetic acid did not change up to week 4 and increased significantly (*p* < 0.05) at week 8. Acetic acid is produced from various substrates in cheese, i.e., lactose, citrate, lactic acid or amino acids, and it is an intermediate product of the metabolic pathways of starter and NSLAB [39,40]. This is in accordance with the significant (*p* < 0.05) effect of the interactions of the experimental factors on the acetic acid concentration in the present experiments. Acetic acid levels were not significantly (*p* > 0.05) affected by salting method, salt content and ripening temperature.

### 3.7. Organoleptic Parameters

All cheese groups got high scores for organoleptic parameters (Table 4). Appearance and texture scored values > 90%, flavor scored values > 85% and the total organoleptic score was > 87%. Flavor was characterized as aromatic and sweet and texture as semi-hard and elastic. The salting method was the sole factor that affected the scores. Brine-salted cheese exhibited a significantly (*p* < 0.05) higher score, on average, for flavor (8.80 ± 0.073), compared to dry-salted cheese (8.57 ± 0.073). Moreover, total organoleptic score was significantly (*p* < 0.05) higher in BS cheese (89.64 ± 0.602) compared to S cheese (89.64 ± 0.602). Salt reduction did not impair the organoleptic parameters, similar to other findings for semi-hard cheese with variable salt contents [30]. No off-flavors and textural defects were detected in reduced-salt cheese, contrasting to other reports for bitter taste and soft texture [31,33]. The increase in ripening temperature at weeks 3 and 4 did not exhibit any significant influence, although it significantly affected proteolysis and NSLAB counts (Section 3.2 and Section 3.5).

## 4. Conclusions

The findings of the present study taken together indicate that salting in brine and increase in the ripening temperature at a particular period of ripening improve textural and organoleptic features of a reduced-fat (≅10.5% fat), sheep milk, semi-hard cheese. Moreover, under these conditions, an impairment of cheese properties and stability due to salt reduction (up to ≅0.5% sodium) does not occur. The application of packaging at an early stage prevents the moisture loss during ripening without affecting cheese attributes. Based on the present findings, new experiments in regard to other cheese types, shapes and sizes can be organized.

## Figures and Tables

**Table 1:** Physicochemical composition (%) during the ripening and storage of sheep milk reduced-fat cheese. S, dry-salted cheese; BS, brine-salted cheese; CS, control-salt (S1, BS1) cheese; RS, reduced salt (S2, BS2) cheese; SE, standard error; S/M, salt in moisture; FDM, fat on dry matter; MNFS, moisture on non-fat substances; P/F, protein-to-fat ratio; PDM, protein on dry matter.

Factors	pH	Acidity ^1^	Moisture ^2^	Salt ^2^	S/M ^2^	Fat ^3^	Protein ^3^	FDM ^3^	MNFS ^3^	P/F ^3^	PDM ^3^
Weeks of ripening											
1	5.11	1.09 ^a^	51.64 ^b^	1.81 ^a^	3.38 ^a^	10.96 ^b^	30.48 ^b^	21.95 ^b^	56.25 ^c^	2.79 ^a^	61.07 ^b^
4	5.09	1.13 ^a^	51.17 ^a^	1.88 ^a^	3.54 ^a^	10.35 ^a^	30.24 ^a,b^	20.77 ^a^	55.98 ^b,c^	2.93 ^b^	60.71 ^b^
8	5.08	1.36 ^b^	51.17 ^a^	1.98 ^b^	3.73 ^b^	10.38 ^a^	30.10 ^a^	20.74 ^a^	55.74 ^a,b^	2.90 ^b^	60.15 ^a^
16	5.08	1.44 ^c^	51.11 ^a^	1.97 ^b^	3.71 ^b^	10.36 ^a^	30.20 ^a,b^	20.65 ^a^	55.60 ^a^	2.92 ^b^	60.22 ^a^
SE	0.015	0.017	0.142	0.029	0.057	0.063	0.110	0.103	0.132	0.017	0.140
Salting method											
S	5.11 ^b^	1.24 ^a^	51.33	2.11 ^b^	3.96 ^b^	10.43 ^a^	30.07 ^a^	20.89 ^a^	55.92	2.89	60.26 ^a^
BS	5.07 ^a^	1.27 ^b^	51.22	1.70 ^a^	3.22 ^a^	10.59 ^b^	30.44 ^b^	21.16 ^b^	55.86	2.88	60.82 ^b^
SE	0.010	0.012	0.100	0.021	0.040	0.044	0.078	0.073	0.094	0.012	0.099
Salt content											
CS	5.10	1.25	50.87 ^a^	2.15 ^b^	4.05 ^b^	10.55	30.40 ^b^	20.96	55.52 ^a^	2.89	60.39 ^a^
RS	5.08	1.26	51.68 ^b^	1.67 ^a^	3.13 ^a^	10.47	30.11 ^a^	21.09	56.27 ^b^	2.88	60.69 ^b^
SE	0.010	0.012	0.100	0.021	0.040	0.044	0.078	0.073	0.094	0.012	0.099
Ripening temperature ^4^											
11 °C	5.09	1.24	51.28	1.91	3.59	10.48	30.23	20.99	55.90	2.89	60.51
18 °C	5.09	1.27	51.27	1.91	3.59	10.53	30.29	21.06	55.88	2.88	60.57
SE	0.010	0.012	0.100	0.021	0.040	0.044	0.078	0.073	0.094	0.012	0.099

^1^ The titratable acidity is expressed as lactic acid (%); ^2^ Estimation was carried out by means of reference methods [12,13,14]; ^3^ Estimation was carried out by means of NIR analyzer; ^4^ Ripening temperature at the 3rd and 4th week of ripening; ^a–c^ Different letters (a–c) within each column group indicate statistically significant differences caused by each experimental factor (LSD, *p* < 0.05).

**Table 2:** Color parameters and meltability (0–10 units, Section 2.2.) during the ripening and storage of sheep milk reduced-fat cheese. S, dry-salted cheese; BS, brine-salted cheese; CS, control-salt (S1, BS1) cheese; RS, reduced salt (S2, BS2) cheese; SE, standard error; L*, lightness (0–100); a*, green vs. red (positive and negative values, respectively); b*, blue vs. yellow (positive and negative values, respectively).

	Color Parameters			Meltability
	L*	a*	b*	
Weeks of ripening				
4	76.53	−2.46	11.66 ^a^	1.28 ^a,b^
8	76.67	−2.47	11.75 ^a^	1.13 ^a^
16	76.81	−2.44	12.36 ^b^	1.40 ^b^
SE	0.300	0.044	0.152	0.064
Salting method				
S	76.19 ^a^	−2.52 ^a^	11.99	1.24
BS	77.16 ^b^	−2.39 ^b^	11.86	1.30
SE	0.245	0.036	0.124	0.053
Salt content				
CS	76.14 ^a^	−2.49	12.00	1.15 ^a^
RS	77.20 ^b^	−2.42	11.84	1.40 ^b^
SE	0.245	0.036	0.124	0.053
Ripening temperature ^1^				
11 °C	76.94	−2.49	11.99	1.26
18 °C	76.40	−2.42	11.85	1.28
SE	0.245	0.036	0.124	0.053

^1^ Ripening temperature at the 3rd and 4th week of ripening; ^a–b^ Different letters (a–b) within each column group indicate statistically significant differences caused by each experimental factor (LSD, *p* < 0.05).

**Table 3:** Residual sugars and organic acids (mg/100 g) during the ripening and storage of sheep milk reduced-fat cheese. S, dry-salted cheese; BS, brine-salted cheese; CS, control-salt (S1, BS1) cheese; RS, reduced-salt (S2, BS2) cheese; SE, standard error.

Factors	Galactose	Lactic Acid	Citric Acid	Orotic Acid	Formic Acid	Acetic Acid
Weeks of ripening						
1	132.6 ^c^	2326.3 ^a^	519.0 ^a^	3.9 ^a^	68.0 ^a^	95.7 ^a^
4	73.5 ^b^	2621.0 ^b^	508.5 ^a^	4.4 ^b^	91.3 ^b^	99.2 ^a^
8	56.3 ^a^	2709.9 ^c^	598.0 ^b^	5.3 ^c^	115.1 ^c^	119.9 ^b^
SE	5.80	19.24	6.15	0.11	3.76	6.90
Salting method						
S	85.7	2506.1 ^a^	545.9	4.6	90.6	105.8
BS	89.2	2598.7 ^b^	537.8	4.5	92.3	104.0
SE	4.91	15.99	5.02	0.09	3.23	5.62
Salt content						
CS	99.3 ^b^	2486.7 ^a^	536.5	4.6	94.9	103.9
RS	75.6 ^a^	2618.0 ^b^	547.2	4.5	88.0	105.9
SE	4.91	15.99	5.02	0.09	3.23	5.62
Ripening temperature ^1^						
11 °C	97.7 ^b^	2524.2 ^a^	528.2 ^a^	4.6	89.6	98.0
18 °C	77.3 ^a^	2580.5 ^b^	555.5 ^b^	4.5	93.4	111.8
SE	4.91	15.99	5.02	0.09	3.23	5.62

^1^ Ripening temperature at the 3rd and 4th week of ripening; ^a–c^ Different letters (a–c) within each column group indicate statistically significant differences caused by each experimental factor (LSD, *p* < 0.05).

**Table 4:** Evolution of organoleptic parameters during the ripening and storage of sheep milk reduced-fat cheese. S, dry-salted cheese; BS, brine-salted cheese; CS, control-salt (S1, BS1) cheese; RS, reduced salt (S2, BS2) cheese; SE, standard error.

Factors	Appearance (1–10)	Texture (1–10)	Flavor (1–10)	Total Score (%) ^2^
Weeks of ripening				
8	9.10	9.08	8.73	89.08
16	9.15	9.03	8.63	88.42
SE	0.064	0.072	0.073	0.602
Salting method				
S	9.14	8.97	8.57 ^a^	87.86 ^a^
BS	9.11	9.14	8.80 ^b^	89.64 ^b^
SE	0.064	0.072	0.073	0.602
Salt content				
CS	9.08	9.02	8.69	88.58
RS	9.17	9.09	8.68	88.92
SE	0.064	0.072	0.073	0.602
Ripening temperature ^1^				
11	9.14	9.05	8.70	88.84
18	9.11	9.06	8.67	88.66
SE	0.064	0.072	0.073	0.602

^1^ Ripening temperature at the 3rd and 4th week of ripening; ^2^ estimated as described in Section 2.2; ^a–b^ Different letters (a–b) within each column group indicate statistically significant differences caused by each experimental factor (LSD, *p* < 0.05).

## Data Availability

Data are presented in the manuscript.

## References

[1] WHO (2023). Total Fat Intake for the Prevention of Unhealthy Weight Gain in Adults and Children: WHO Guideline.

[2] WHO (2012). Guideline: Sodium Intake for Adults and Children.

[3] Guinee T.P., McSweeney P.L.H., Fox P.F., McSweeney P.L.H. (2006). Significance of milk fat in cheese. Advanced Dairy Chemistry. Lipids.

[4] Johnson M.E., Kapoor R., McMahon D.J., McCoy D.R., Narasimmon R.G. (2009). Reduction of sodium and fat levels in natural and processed cheeses: Scientific and technological aspects. Compr. Rev. Food Sci. Food Saf..

[5] Khanal B.K.S., Bansal N., Truong T., Lopez C., Bhandari B., Prakash S. (2020). Dairy fat replacement in low-fat cheese (LFC): A review of successful technological interventions. Dairy Fat Products and Functionality.

[6] Mistry V.V. (2001). Low fat cheese technology. Intern. Dairy J..

[7] Banks J.M. (2004). The technology of low-fat cheese manufacture. Intern. J. Dairy Technol..

[8] Bansal V., Mishra S.K. (2020). Reduced-sodium cheeses: Implications of reducing sodium chloride on cheese quality and safety. Compr. Rev. Food Sci. Food Saf..

[9] Cruz A.G., Faria J.A.F., Pollonio M.A.R., Bolini H.M.A., Celeghini R.M.S., Granato D., Shah N.P. (2011). Cheeses with reduced sodium content: Effects on functionality, public health benefits and sensory properties. Trends Food Sci. Technol..

[10] Fox P.F., Guinee T.P., Cogan T.M., McSweeney P.L.H. (2000). Fundamentals of Cheese Science.

[11] Guinee T.P., Fox P.F., Fox P.F., McSweeney P.L.H., Cogan T.M., Guinee T.P. (2004). Salt in cheese: Physical, chemical and biological aspects. Cheese: Chemistry, Physics and Microbiology, General Aspects.

[12] (2008). Milk-Determination of Fat Content.

[13] (2004). Cheese and Processed Cheese—Determination of the Total Solids Content (Reference Method).

[14] (2006). Cheese and Processed Cheese Products—Determination of Chloride Content—Potentiometric Titration Method.

[15] Moschopoulou E., Sakkas L., Zoidou E., Theodorou G., Sgouridou E., Kalathaki C., Liarakou A., Chatzigeorgiou A., Politis I., Moatsou G. (2018). Effect of milk kind and storage on the biochemical, textural and biofunctional characteristics of set-type yoghurt. Intern. Dairy J..

[16] Gunasekaran S., Ak M.M. (2003). Cheese Rheology and Texture.

[17] Moatsou G., Zoidou E., Choundala E., Koutsaris K., Kopsia O., Thergiaki K., Sakkas L. (2019). Development of reduced-fat, reduced-sodium semi-hard sheep milk cheese. Foods.

[18] Park J., Rosenau J.R., Peleg M. (1984). Comparison of four procedures of cheese meltability evaluation. J. Food Sci..

[19] Sakkas L., Alatini E., Moatsou G. (2021). Use of sweet sheep buttermilk in the manufacture of reduced-fat sheep milk cheese. Intern. Dairy J..

[20] (2003). Yogurt-Enumeration of Characteristic Microorganisms-Colony-Count Technique at 37 Degrees C.

[21] Lepesioti S., Zoidou E., Lioliou D., Moschopoulou E., Moatsou G. (2021). Quark-type cheese: Effect of fat content, homogenization, and heat treatment of cheese milk. Foods.

[22] FAO, WHO (2011). Codex Alimentarius International Food Standards: General Standard for Cheese CXS 283-1978.

[23] Walstra P., Vouters J., Geurts T. (2006). Dairy Science and Technology.

[24] Salaün F., Mietton B., Gaucheron F. (2005). Buffering capacity of dairy products. Intern. Dairy J..

[25] Hassan A., Johnson M.E., Lucey J.A. (2004). Changes in the proportions of soluble and insoluble calcium during the ripening of Cheddar cheese. J. Dairy Sci..

[26] Upreti P., Metzger L.E. (2007). Influence of calcium and phosphorus, lactose, and salt-to-moisture ratio on Cheddar cheese quality: pH changes during ripening. J. Dairy Sci..

[27] Fox P.F., Cogan T.M., Fox P.F., McSweeney P.L.H., Cogan T.M., Guinee T.P. (2004). Factors that affect the quality of cheese. Cheese: Chemistry, Physics and Microbiology. General Aspects.

[28] Soodam K., Ong L., Powell I.B., Kentish S.E., Gras S.L. (2017). Effect of elevated temperature on the microstructure of full fat Cheddar cheese during ripening. Food Struct..

[29] Akkerman L., Søndergaard Kristensen L., Jespersen L., Balling Ryssel M., Mackie A., Larsen N.N., Andersen U., Nørgaard M.K., Løkke M.M., Møller J.R. (2017). Interaction between sodium chloride and texture in semi-hard Danish cheese as affected by brining time, DL-starter culture, chymosin type and cheese ripening. Intern. Dairy J..

[30] Sihufe G.A., De Piante Vicín D.A., Marino F., Ramos E.L., Nieto I.G., Karlen J.G., Zorrilla S.E. (2018). Effect of sodium chloride reduction on physicochemical, biochemical, rheological, structural and sensory characteristics of Tybo cheese. Intern. Dairy J..

[31] Søndergaard L., Ryssel M., Svendsen C., Høier E., Andersen U., Hammershøj M., Møller J.R., Arneborg N., Jespersen L. (2015). Impact of NaCl reduction in Danish semi-hard Samsoe cheeses on proliferation and autolysis of DL-starter cultures. Intern. J. Food Microbiol..

[32] Wilkinson M.G., Guinee T.P., O’Callaghan D.M., Fox P.F. (1994). Autolysis and proteolysis in different strains of starter bacteria during Cheddar cheese ripening. J. Dairy Res..

[33] Dugat-Bony E., Bonnarme P., Fraud S., Catellote J., Sarthou A.S., Loux V., Rué O., Bel N., Chuzeville S., Helinck S. (2019). Effect of sodium chloride reduction or partial substitution with potassium chloride on the microbiological, biochemical and sensory characteristics of semi-hard and soft cheeses. Food Res. Intern..

[34] Møller K.K., Rattray F.P., Høier E., Ardö Y.M. (2012). Manufacture and biochemical characteristics during ripening of Cheddar cheese with variable NaCl and equal moisture content. Dairy Sci. Technol..

[35] Rulikowska A., Kilcawley K.N., Doolan I.A., Alonso-Gomez M., Nongonierma A.B., Hannon J.A., Wilkinson M.G. (2013). The impact of reduced sodium chloride content on Cheddar cheese quality. Intern. Dairy J..

[36] Califano A.N., Bevilacqua A.E. (2000). Multivariate analysis of the organic acids content of Gouda type cheese during ripening. J. Food Compos. Anal..

[37] Mullin W.J., Emmons D.B. (1997). Determination of organic acids and sugars in cheese, milk and whey by high performance liquid chromatography. Food Res. Intern..

[38] Ong L., Shah N.P. (2009). Probiotic Cheddar cheese: Influence of ripening temperatures on survival of probiotic microorganisms, cheese composition and organic acid profiles. LWT-Food Sci. Technol..

[39] Upreti P., McKay L.L., Metzger L.E. (2006). Influence of calcium and phosphorus, lactose, and salt-to-moisture ratio on Cheddar cheese quality: Changes in residual sugars and water-soluble organic acids during ripening. J. Dairy Sci..

[40] McSweeney P.L.H., Sousa M.J. (2000). Biochemical pathways for the production of flavour compounds in cheeses during ripening: A review. Lait.

[41] Michel V., Martley F.G. (2001). Streptococcus thermophilus in Cheddar cheese—production and fate of galactose. J. Dairy Res..

